# Developing State Leadership in Maternal and Child Health: Process Evaluation Findings from a Work-Based Learning Model for Leadership Development

**DOI:** 10.1007/s10995-022-03444-z

**Published:** 2022-04-30

**Authors:** Karl E. Umble, Laura Powis, Alexandria M. Coffey, Lewis Margolis, Amy Mullenix, Hiba Fatima, Stephen Orton, W. Oscar Fleming, Kristen Hassmiller Lich, Dorothy Cilenti

**Affiliations:** 1 Department of Health Policy and Management, University of North Carolina - Chapel Hill, 113 Rosenau Hall CB #7411, Chapel Hill, NC 27599-7411 USA; 2 The Association of Maternal and Child Health Programs, 1825 K Street Suite 250, Washington, DC 20006-1202 USA; 3 Department of Maternal and Child Health, University of North Carolina - Chapel Hill, 135 Dauer Drive, Chapel Hill, NC 27599 USA; 4 The National MCH Workforce Development Center, University of North Carolina - Chapel Hill, 135 Dauer Drive, Chapel Hill, NC 27599 USA; 5 North Carolina Institute for Public Health, University of North Carolina - Chapel Hill, 135 Dauer Drive, Chapel Hill, NC 27599 USA; 6 National Implementation Research Network, University of North Carolina - Chapel Hill, Campus, Box 8180, Chapel Hill, NC 27516 USA; 7 Department of Health Policy and Management, University of North Carolina - Chapel Hill, 135 Dauer Drive, Chapel Hill, NC 27599 USA

**Keywords:** Maternal and child health, Workforce, Leadership, Program evaluation

## Abstract

**Objectives:**

Since 2013 the MCH Bureau has supported the National MCH Workforce Development Center to strengthen the Title V MCH workforce. This article describes the Center’s Cohort Program and lessons learned about work-based learning, instruction, and coaching.

**Description:**

The Cohort Program is a leadership development program that enrolls state-level teams for skill development and work-based learning to address a self-identified challenge in their state. Teams attend a Learning Institute that teaches concepts, skills, and practical tools in systems integration; change management and adaptive leadership; and evidence-based decision-making and implementation. Teams then work back home on their challenges, aided by coaching. The Program’s goals are for teams to expand and use their skills to address their challenge, and that teams would strengthen programs, organizations, and policies, use their skills to address other challenges, and ultimately improve MCH outcomes.

**Methods:**

This process evaluation is based on evaluation forms completed by attendees at the three-day Learning Institute; six-month follow-up interviews with team leaders; and a modified focus group with staff.

**Results:**

Participants and staff believe the Cohort Program effectively merges a practical skill-based curriculum, work-based learning in teams, and coaching. The Learning Institute provides a foundation of skills and tools, strengthens the team’s relationship with their coach, and builds the team. The work-based learning period provides structure, accountability, and a “practice space” for teams to apply the Cohort Program’s skills and tools to address their challenge. In this period, teams deepen collaborations and often add partners. The coach provides accessible and tailored guidance in teamwork and skill application. These dimensions helped teams in develop skills and address state-level MCH challenges.

**Conclusions for Practice:**

Continuing professional development programs can help leaders learn to address complex state-level MCH challenges through integrated classroom-based skills development, work-based learning on state challenges, and tailored coaching.

## Significance

*What is already known about this subject?* Prior studies have described public health team-based leadership development programs that combine instruction, team work-based learning, and coaching. Such programs can strengthen participants’ knowledge, skills, confidence, practices, and networks and help them improve programs, organizations, systems, and policies.

*What this study adds* This study provides more detail about how interactive instructional methods, extensive work-based learning in teams, tailored coaching, and an evidence-based curriculum can help leaders learn to address complex state-level MCH challenges, including how learners experience the benefits of these methods.

## Introduction

To improve public health (PH) today, leaders must develop partnerships and steer performance improvement at organizational and system levels (Erwin & Brownson, 2017; Public Health Leadership Forum, 2020). Needs assessments have identified leaders’ skill gaps in related areas, including building partnerships, integrating systems, collaborating with diverse populations, leading change, solving problems, engaging with policy makers, and using data to gauge needs and progress (Bogaert et al., 2019; Kaufman et al., 2014; National Consortium for PH Workforce Development, 2017; Sellers et al., 2015).

Maternal and child health (MCH) studies have concurred. A 2008 report identified systems thinking, change management, and general management as skills that MCH professionals needed to develop (AMCHP, 2008). A 2016 study found that MCH leaders wanted more training in building systems, managing change, and evidence-based PH (AMCHP, 2016). The MCH Leadership Competencies (USDHHS, 2018) have supported developing leaders who can strengthen systems and lead organizational change, as have other needs assessments and statements (Grason et al., 2012; Kavanagh, 2015; Petersen, 2015).

In response, the federal MCH Bureau (MCHB) has supported the National MCH Workforce Development Center since 2013. Headquartered at the University of North Carolina at Chapel Hill and with academic and practice partners around the nation, the Center offers training and development programs to equip the Title V workforce to meet today’s challenges and transform organizations and systems (Clarke & Cilenti, 2018; Handler et al., 2018; Margolis et al., 2017). The Center works closely with the MCHB and the Association of Maternal and Child Health Programs (AMCHP).

This article describes the Center’s flagship Cohort Program (henceforth, “the Cohort Program”), a 6–8-month leadership development program that enrolls state-level teams and includes work-based learning to address a self-identified MCH-related problem at the state level (henceforth, "challenge”). The article then describes what the Center has learned about how to structure the Cohort Program to improve “collaborative” or “shared" leadership skills.

The Cohort Program builds on wider calls for professional development to use multiple learning methods over time to enable professionals to improve their workplace performance (Davis et al., 1999; Institute of Medicine, 2010; Moore et al., 2009). By expanding classroom learning into the workplace, work-based learning fosters learning from work practices, typically with others engaged in similar work (Raelin, 2006, 2019). The Cohort Program’s work-based learning process may more specifically be named as “action learning,” in which participants formulate and take action on a challenge and reflect on what they learn from their experience, aided by peers and coaches (Raelin, 2006, 2019). Prior evaluations have shown that work-based learning in teams can improve skills and confidence, strengthen collaboration, and help participants improve programs, organizations, systems, and policies (Orton et al., 2006; Umble et al., 2006, 2009, 2011a, 2011b, 2012).

This article also provides a case study of a program that develops “collaborative” or “shared” leadership skills through enrolling teams to address multi-party challenges (Edmonstone et al., 2019; Raelin, 2019). Scholars have increasingly described leadership as a social process of dialog, action, and reflection among stakeholders which leads to shared direction, alignment of people and units around shared goals, and commitment (McCauley & Fick-Cooper, 2015). For example, Heifetz, Linsky, and others have described “adaptive” leadership (Heifetz et al., 2009) as a form of leadership needed when organizations and systems face complex challenges that can only be addressed if the stakeholders adapt, or learn new ways of addressing challenges together. In adaptive leadership, individual leaders do not prescribe technical answers to challenges, but instead foster a shared process in which stakeholders reflect, surface conflicts, and work together to address the challenges.

This article builds on two prior published evaluations of the Cohort Program. In Margolis et al. (2017), the first cohort reported that the program had strengthened skills and partnerships that they could use to advance work toward Title V program goals. Clarke and Cilenti (2018) found that despite several barriers to sustained collaborative work, leaders and teams from the initial cohort were using key skills taught.^1^

The Office of Human Research Ethics at the University of North Carolina at Chapel Hill reviewed this evaluation and determined it was exempt from IRB approval.

## Cohort Program Overview

**Fig. 1:**
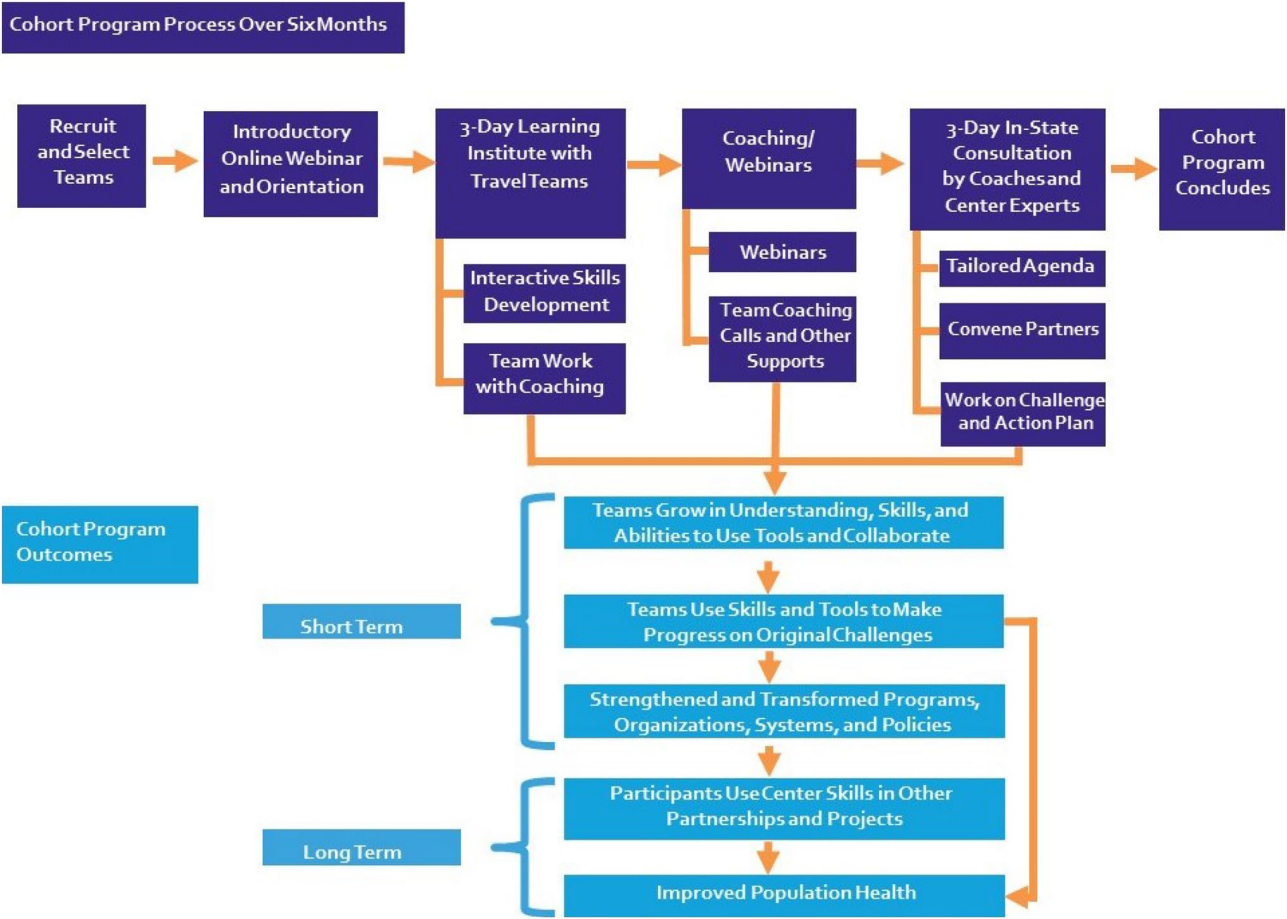
How the cohort program improves practice and the public’s health

The Cohort Program (Fig. 1) enrolls state-level multisector teams to learn new skills and practice applying them on a state-selected challenge related to health transformation^1^. As defined by the Center, “Health transformation shifts the emphasis… from disease management to prevention and population health management, while improving access to affordable health care; utilizes an interprofessional/interdisciplinary approach; integrates primary care, specialty care and PH; develops evidence-based, efficient health systems that better incorporate ongoing quality improvement; and drives partnerships across sectors to optimize the wellbeing of MCH populations” (Margolis et al., 2017, pp. 2001–2002).

### Objectives

The Cohort Program’s objectives (Fig. 1) are:

1. To help teams grow in understanding, skills, and abilities to use Center-taught tools to frame MCH challenges and collaborate to address them.
2. To support teams’ use of Center-taught skills and tools to address their original challenge in a manner that is systematic, adaptive, grounded in evidence, ambitious, and aimed toward improving team-selected outcomes related to MCHB Title V outcome measures.
3. Through teams’ work, to strengthen and transform PH programs, organizations, systems, and policies.
4. To equip participants to use Center skills with subsequent challenges and partnerships.
5. Ultimately, to improve the public’s health, including MCH populations served by Title V programs.

### Teams

The full state team, or “back-home” team, typically has 10–20 members. The majority are leaders and staff members from the state agency funded by the federal Title V MCH Block Grant Program.^2^ Other team members have mainly worked for other state health agencies, local health departments, or partners such as patient advocacy organizations, professional associations, and health systems. The Center encourages states to include members representing the groups and organizations that can effectively and systemically address the team’s challenge. An average of 6.2 agencies have been represented on each team; teams have enrolled from all regions of the United States (Table 1). Through an interview, the Center explores each proposed team’s membership and capacity to address their challenge so changes can be made prior to the launch. The back-home team later selects a “travel team” of five or six members to travel to Chapel Hill, North Carolina for the Learning Institute. MCHB funding pays for all team expenses, including travel.

**Table 1:** Descriptive data for state teams participating in the Cohort Program between 2014 and 2020 (N = 53 teams)

Characteristic	Number of teams^a^	Percentage of teams (%)
U.S. Region		
Northeast	7	13
Midwest	14	26
West	9	17
South	23	43
Average number of agencies represented in back-home teams^b^	6.2	

^a^Some states completed the Cohort Program more than once; therefore, they are counted more than once in the table

^b^One state did not supply the number of agencies represented on their team

### Cohort Program Structure and Curriculum

The Cohort Program has two main components: (1) in-person Learning Institute (LI) and (2) post-LI support. Its curriculum centers on three interrelated skills (Table 2). Systems Integration skills help participants understand how systemic forces interact to produce and reinforce challenges, identify actors to address them, and develop shared solutions. Change Management teaches teams to use health transformation and adaptive leadership concepts and includes segments on partnerships and team development. Evidence-based Decision-Making and Implementation teaches participants how to select, adapt, and sustainably implement evidence-based practices and innovations. The Cohort Program emphasizes health equity and supports family engagement in service and policy development.

**Table 2:** Cohort Program curriculum topics

General curriculum topic	Concepts and skills taught
Systems integration	Introduce and motivate systems thinking
	Methods and tools to facilitate cross-stakeholder discussion of the situation of interest (i.e., What motivated teams to apply)
	Methods and tools to develop a shared understanding of the focal challenge and identify leverage points that help shift the entire system and not simply treat the "symptom^−^ of the problem”
	Understand the network of stakeholders that are needed For an initiative (e.g., who they are, what they care about, how to get the right people engaged clarify roles and responsibilities)
	Understand and work to strengthen your team and/or initiative as a system
Change management and adaptive leadership	Health transformation concepts
	Connection between change, health transformation, and team challenge
	Building teams for healthy transformation
	Mutual Learning Model
	Technical vs adaptive leadership
	Building and sustaining partnerships
Evidence-based decision- making and implementation	Implementation stages
	Developing and using performance indicators
	Understanding purpose and scope of evidence-based decision making and implementation
	Methods and tools to support implementation team development effective communication and continuous learning
	Assess implementation practice to identify strengths and opportunities for improvement
Heath equity	Foundational practice for healthy equity
	Health equity in transformational work
Supporting family partnership	Support for family partners participating in Cohort Program via peer support groups
	Family engagement in system toolkit
	Standards of quality for family strengthening and support tool

### Learning Institute

The Cohort Program begins with an online webinar (Fig. 1) followed by a three-day Learning Institute (LI) in Chapel Hill, North Carolina for the travel team. The LI includes skill-development workshops and extensive time with the team’s assigned coach, who helps them apply skills and tools to their challenge and connects them with other Center experts and resources. While this evaluation includes only teams that completed the program before the pandemic occurred, the Cohort Program moved to virtual delivery during the COVID-19 pandemic but largely retained its curriculum and instructional strategies. The virtual LI taught fewer concepts, but coaches added more one-on-one learning sessions with their teams, which partly made up for the reduced content and further tailored the learning to the teams.

To foster practice change, the Cohort Program teaches teams to use applied “tools” that help them practice concepts and skills (Table 3). The facilitator (instructor) explains what each tool accomplishes and demonstrates through examples how teams can use it. Teams then apply the tool to their challenge, aided by the facilitator and their coach. For example, in the module on systems thinking, the facilitator teaches the causal loop diagram and shares examples. Teams then build a causal loop diagram for their challenge. The tools thus help participants move from “knowledge about” key concepts to workplace performance (Institute of Medicine, 2010), and enable them to teach the tools to others back home.

**Table 3:** Explanation of center curriculum tools

Tools to develop the team	
Core conversations	This tool guides groups through a series of questions to explore multiple aspects of their work together, including strengths, dissent, and commitment to the work
Conversational sweet spot	This tool helps individuals balance condor and curiosity in conversations to promote positive relationships with colleagues
Coat of arms	This creative activity relies on individual and team strengths to produce a visual representation of a team’s transformation challenge
Network map	This tool allows individuals to visually represent the strength and density of stakeholder relationships that are currently or could be leveraged to support team efforts
System support map	A deep-dive mapping exercise to depict and individual’s responsibilities, needs, experience with available resources, and wishes. It can be used to describe consumer needs, team success, or define and strengthen an MCH system/initiative
Tools to understand the challenge	
5Rs	A conversation guide to describe the system individuals work in by depicting diverse perspectives about success, roles, resources to support change, and rules and relationships that must be understood or changed to improve outcomes
Individual challenge statements	A structured exercise that prompts teams to capture diverse perspectives on a complex challenge, allowing team members to understand stakeholder priorities and vocabulary
Group challenge statement	This tool consolidates multiple individual challenge statements into a consensus document
Aim statement	Often building on a Group Challenge Statement, as aim statement provides a concise description of project goals and vision, clearly stating time frame and perspective
Casual loop diagram	Casual loop diagrams are used to elicit and integrate mental models, examine root cause of challenges, and identify key leverage points for action
Tools to consider action steps	
Implementation staging	A frame to examine the “life course” of a complex effort and identify action steps related to each implementation stage
Synthesize the evidence	This tool can be used to organize and summarize key information/findings from your search for “what works.”
Key driver diagram	A visual summary of the overall strategy that illustrates pathway of challenge and priority focus areas
Implementation support checklist	A checklist to prompt consideration of the organizational, leadership, and competency supports
Tools to document and communicate	
Team roaster	Team rosters are used to clarify roles in complex projects. Implementation team roasters are used to document roles of individuals on implementation teams
Communication protocol	A tool to document agreements with stakeholders with whom the team needs to share information and from whom the team needs information
30/30	A tool to track the progress and learning of the team

### Post-LI Support

After the LI, travel teams return home to advance work on their challenge, together with their larger back-home team. Teams follow a structured approach, including devising a logic model and attending supplemental webinars to teach additional skills or tools. Coaches continue to work with their teams by telephone, video, and email to provide feedback on products, advice on team process and tasks, and referrals to Center experts. The coach and other Center experts visit each state for a multi-day tailored in-state consultation in which they help the team and other stakeholders to apply skills and tools to their challenge. At the Cohort Program’s concluding webinar, teams celebrate progress and describe next steps.

Between 2014 and 2020, the Center offered the Cohort Program eight times to 53 teams (averaging 6.5 teams per cohort). Frequent team goals have included improving services for Children and Youth with Special Health Care Needs (CYSHCN) (25% of teams), building systems to improve services spanning multiple populations or topics (19%), improving child health (10%), improving adolescent health, women’s health, developmental screening, and family engagement in MCH (8% each), among others. To address these topics, teams have used Center skills to strengthen partnerships (19%), enhance service delivery (17%), strengthen and streamline screening systems (15%), strengthen health systems (15%), and enhance care coordination (12%), among other objectives. Box 1 briefly describes two example challenges.

Box 1: Example Team Challenges **Expanding Use of Fluoride Varnish**

**Team** Leaders from the state’s Medicaid, Oral Health, and Perinatal and Women’s Health units and the state’s Children’s Health Insurance Program (CHIP).

**Team challenge** Update the state’s Children’s Health Insurance Program (CHIP) reimbursement policies to align with state Medicaid policies. Communicate the state Medicaid’s new policy of expanded coverage for fluoride varnish reimbursement to stakeholders, including medical and dental providers, in order to expand fluoride varnish use and ultimately reduce Early Childhood Caries.

**Approach to addressing the challenge** The team used Center skills and tools, such as system support mapping and action planning, to plan how they would tell providers about the expanded varnish coverage and new billing procedures. The Cohort program enhanced the team’s strategic thinking and relationship-building skills, leading to many successes.

**Results** The team celebrated two major accomplishments:

1. Updated the state’s CHIP coverage policy to align with the State’s Medicaid policy.
2. Surveyed nearly 180 providers statewide to assess their awareness and practices related to American Academy of Pediatric fluoride varnish recommendations. Used findings to identify opportunities for continuing education and referral systems.

**Improving Health Equity**

**Team** Department of Health unit leaders from the state’s Bureau of Family Health (BFH), Office of Health Equity, and Office of the Deputy Secretary for Health Promotion and Disease Prevention.

**Team challenge** Build health equity skills of BFH staff so that staff can improve internal BFH progress on health equity and help BFH vendors develop health equity plans, ultimately improving MCH outcomes through better serving underserved communities.

**Approach to addressing the challenge** The team used Center skills and tools to address their challenge, such as the 30/30, 5R’s, causal loop diagramming, measurement tables, and appreciative inquiry. These tools helped the team plan, engage with BFH employees and stakeholders, and measure progress.

**Results** The team developed a health equity technical assistance document for internal administrators, implemented a BFH employee survey about health equity, and organized.

BFH sub-teams. They developed a three-year work plan for the BFH Health Equity committee and began helping their vendors and grantees incorporate equity concepts into their work.

## Methods

This process evaluation is based on data from the following sources.

### Post-learning Institute Evaluation Forms

At the LI's conclusion, travel team members complete an evaluation form that captures the most and least helpful aspects of the LI. Of 190 participants in the first five cohorts, 165 (86%) completed the form. Qualitative data from these evaluations from the first five cohorts were reviewed and categorized using inductive coding methods, with codes emerging from individual responses.

### Six-Month Follow-Up Interviews with Team Leaders

A Center evaluator interviewed one self-selected team leader from each of the teams in the first five cohorts; a few states included a second team member in the interview. Interviews focused on this evaluation question: How did the Cohort Program help them advance their MCH population health goals through their team challenge? The interview guide included multiple choice, listing, and open-ended questions. Respondents were encouraged to circulate the guide to the other team members to solicit their input. The 32 interviews each lasted approximately one hour and were recorded and transcribed. Evaluators used ATLAS.ti software to code and analyze the transcripts using deductive codes based on the Cohort Program’s objectives and inductive codes that emerged from the interviews. The evaluators used the “Sort and Sift, Think and Shift” qualitative analysis approach developed by ResearchTalk, Inc. (Maietta et al., 2019).

### Modified Focus Group with Workforce Development Center Staff

After the fifth cohort, the Center’s leadership team led a modified focus group discussion with most members of the Center team (approximately 30 participants). A combination of small group discussions and a summative full group discussion were used to promote candid feedback and provide everyone with adequate opportunity to share their thoughts. Both small and summative group discussions focused on these questions: (1) What is most effective about our current approaches? (2) What are the primary tasks of the workforce we serve? How is the Center helping them get their tasks done? (3) How can we improve? The evaluation team recorded the responses, and then coded and analyzed them for major themes. For this paper, the evaluators used the data and themes that pertained to the Cohort Program.

## Results

**Fig. 2:**
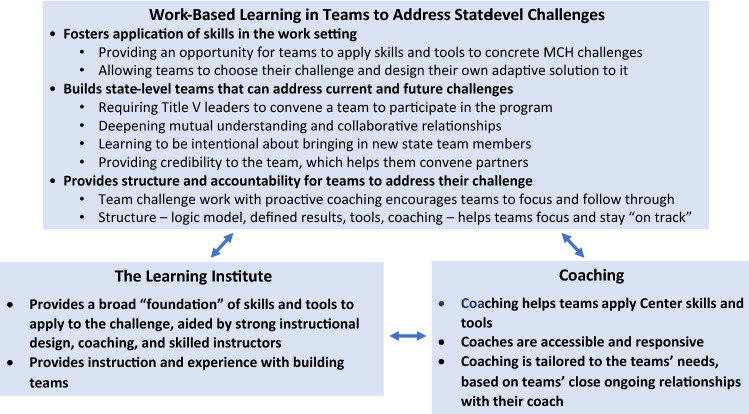
Benefits and helpful features of cohort program components

We present insights from participants and staff about how the Cohort Program’s structure and processes help it accomplish its objectives, in three categories: Work-Based Learning in Teams; the Learning Institute; and Coaching (Fig. 2).

### Theme 1. Work-Based Learning in Teams to Address State-Level Challenges

Work-based learning requires teams to apply new skills to concrete challenges and provides structures and accountability for doing so, while also building their teams.

#### Theme 1.1. Work-Based Learning in Teams Fosters Application of Skills in the Work Setting

##### Providing an Opportunity for Teams to Apply Concepts, Skills and Tools to Concrete MCH Challenges

One learner stated that the Cohort Program provided a “practice space” to progress from understanding a concept or skill to using it with a real challenge. This allows the skills and tools to “come to life” and become part of the leader’s active repertoire:

I’ve had implementation science as part of a college … program but being able to actually have the didactic … [along] with the real live world problem just helped it come to life.

##### Allowing Teams to Choose Their Challenge and Design Their Own Adaptive Solution to It

The Cohort Program’s “practice space” allows each team to choose a challenge and design its adaptive solution to it. These participants noted:

…[T]he Center differs [from other training programs] in the sense that it was completely state-driven in terms of what projects [challenges] we wanted to work on.

You all approve the project and then you let us drive the project. You gave us tools that we needed to drive the project, but we were the actual drivers on the project.

Other participants emphasized the benefits of the Cohort Program’s requirement for the team to reflect on a difficult challenge and select, integrate, and apply skills to develop creative solutions. This is central to the Cohort Program’s emphasis on teaching teams to use “adaptive leadership” to develop tailored “adaptive solutions” instead of relying on outside experts or simple “technical solutions” already in their repertoire. Participants referred to this benefit as making them “sit down and think … outside the box” and giving them the “time where we could really explore the mutual learning [model] and use tools to think about things and be creative.” In this quotation, “mutual learning” refers to a teamwork model that encourages members to listen to others’ reasoning to construct shared solutions, rather than trying to win the argument. Thus, these benefits—developing adaptive solutions through mutual learning—flow from critically reflecting on a challenge as a team:

You guys didn’t give us a magic pill or a magic answer. You gave us tools to help us critically think through … this project but then [also apply] those tools to help us critically think and tease-out other complex problems that we had.

Center staff concurred, noting that teams learn to apply conceptually difficult “skills and concepts to specific MCH contexts, to make them more real and applicable to Title V,” and that “learning happens in the context of hard, often adaptive Title V challenges being addressed.” In this way the Cohort Program “supports actual implementation practice” and systems strengthening “around something states are already struggling with.” This is aided by Center staff “stick[ing] with folks over time,” often enabling the staff to see organizational or practice improvements by the Cohort Program’s end. Staff said the Cohort Program “teaches people to fish” and participants “feel empowered rather than overwhelmed” in addressing systemic challenges.

#### Theme 1.2. Work-Based Learning in Teams Builds State-Level Teams That Can Address Current and Future Challenges

Work-based learning also strengthened teams’ ability to collaborate to address challenges.

##### Requiring Teams

One way the Cohort Program strengthened teams was simply by requiring them. One participant said that the Cohort Program “made us come together to focus on a population health initiative.” Another noted that while their team members—as staff of state agencies—had worked together to some degree beforehand, the Cohort Program “provid[ed] the opportunity formally through the initial [LI and] through the site visit, for us to be brought together and be able to spend … a few days … together. I think that was really valuable because we—all of us—move at such a rapid pace.”

##### Deepening Mutual Understanding and Collaborative Relationships

The Cohort Program also built teams through LI activities that fostered mutual understanding and collaboration. One participant explained that although their team previously “kind of knew each other,” convening for the LI was “kind of like a golden age where you’re in the incubator” and that the “personal time we had in Chapel Hill really sealed the deal.” As a result, this participant explained that they now “automatically” take the perspective of their teammates and consider the entire “system” when reflecting on their team challenge:

[W]henever I talk about home visiting, I automatically think of early intervention. I mean… we remember each other more. We’re more intentional in terms of how we talk about the system…. I think that was transformative as well.

Others also described team building at the LI as beneficial:

[What] our team… found most helpful was the actual team building…. There are a couple of partners who … work quite a distance from us and having that time together really built those relationships, really kind of instilled more trust among those partners.

[The] in-person training was really great…. it really did help us kind of bring together a group of stakeholders throughout the state to do this work and really align the efforts, which was the goal of the project.

##### Learning to be Intentional About Bringing in New State Team Members

One participant noted, “Just being intentional [in bringing in new team members at the LI] really kind of refocused us on being intentional… when we have new people come into [the team]—how we kind of integrate them into that process….” Another noted that the LI had given them “the capacity to do this work and [learn] how to bring people together to do this work…. By being able to bring us together and create the partnerships that we’ve had has really created a trust in this work that people are doing in breastfeeding.”

##### Providing Credibility to the Team, Which Helps Them Convene Partners

One participant noted, “The Center really provided us a lot of credibility.… For our MCO’s [Managed Care Organizations], for our other partners… the Center gave us that credibility that helped to bring them to the table.” Another said the Cohort Program had helped them convene a large team that enhanced the credibility of their deliverable:

[W]hat [the Program] really did allow for our team to do is when we came out with our finished product and our plan, we had an entire statewide team that was on board with the vision and … we had a united front within ourselves… [W]ithout the Center being there to help be that facilitator, we would not have, we wouldn’t be where we are now.

Center staff agreed that the Cohort Program helps “states partner across organizational/disciplinary lines” and “strengthens collaboration with other agencies [and] groups” because it “provide[s] a reason to interact, build relationships, and tools/coaching to support effective relationship-building.” Another staff member said this added credibility in building partnerships gives the Title V agency additional influence and authority, placing its leaders in the center of efforts in which they might otherwise be sidelined.

#### Theme 1.3. Work-Based Learning in Teams Provides Structure and Accountability for Teams to Address Their Challenge

One participant said the Cohort Program “forced us to set aside time and really focus on … these specific activities which is sometimes what you need to do,” while another said the “Center was helpful in keeping us on task and making sure that we followed through.” One attributed this to the Center’s structured approach, noting that “there’s so much value to doing … this structured work. It really … helps with accountability. It moves our project along. Whenever we start deviating, we can always go back to like the intent of the project and look at our tools and the logic model and get back on track.” Another attributed accountability to the proactive work of the coach, who helped keep the work “front and centered.” Another commented:

[T]his [program] was more so really about accountability and keeping us moving forward… focused on results… which is something that we needed.... [M]ost … [training programs] you go to are usually for like a day and then you’re done. People take the training and they throw it on a shelf and they move on but when you have somebody that interacts you with people a little while, it’s kind of hard to just let it drop.

### Theme 2. The Learning Institute

The LI provided skills along with instruction and experience with building teams and an opportunity to learn about other teams’ work.

#### Theme 2.1. The Learning Institute Provides a Broad “Foundation” of Skills and Tools to Apply to the Team Challenge, Aided by Strong Instructional Design, Coaching, and Skilled Facilitators.

When asked to explain how they found the LI useful, some respondents described learning to use all the Cohort Program’s skills and tools. Others mentioned specific skills:

The tools like the system support map helped us understand one another’s responsibility and expectations.

When asked to describe the LI’s most effective aspects, many participants noted its instructional design, which involves presenting a concept or skill along with an applied tool that will help the team use it in practice; demonstrating with an example how the tool may be used; and “team time” to apply the tool to the team challenge with the coach’s help. One participant said it was helpful to “*[take] tools to team time and using them to further define our project aims, goals, objectives, and outcomes.”*

#### Theme 2.2. The Learning Institute Provides Instruction and Experience with Building Teams

As we discussed fully above, the Cohort Program requires that teams enroll and collaborate intensively at the LI to address their challenge. The LI also teaches them to use several tools specifically designed to build teams. In these ways, the LI strengthens leaders’ capacity to build professional teams.

### Theme 3. Coaching

Learners also reported that the Cohort Program’s accessible and tailored coaching was invaluable in helping them apply skills and tools to their work.

#### Theme 3.1. Coaching Helps Teams Apply Center-Taught Skills and Tools

Some participants also said that while the LI provided a “backbone” or “orientation and foundation,” their new skills and tools were deepened by the subsequent coaching to address their challenge.

[The LI] gave us the backbones of what we can do. But then when … [the] coaches came [for the site visit] - I felt like it was then, “Okay now we can apply this to what we’re doing.”

I think coaching was 90 percent of the [Program] experience. I mean, the [3-day] meeting in North Carolina was a good orientation and foundational piece of it, but I think that the coaching was the primary part that was helpful.

#### Theme 3.2. Coaches are Accessible and Responsive

Many participants noted that coaches' accessibility and responsiveness helped teams make progress on their challenges:

I felt like I had great support during the past several months. You know, I could always call up [the coach] and ask questions, kind of talk through some ideas.

#### Theme 3.3. Coaching is Tailored to the Teams’ Needs, Based on Teams’ Close Ongoing Relationships with Their Coach

Participants appreciated the coaches’ tailored guidance:

[In the Program] you have a coach that seems dedicated to your team… It’s not one size fits all. It seems very tailored.

The difference between [other training] and the workforce Center was we developed really close relationships with the people from the workforce Center and it just felt like, “We know these people or they know us” and we’re comfortable in saying “You know, this is what we have right now, this is the problem. How can we resolve this? Can you help us?” - without trying to hide anything.

Having [our coach] sit at our table [at the LI] the entire time, work with us, know where we started from, having the calls and the coaching, [helped us] know we’re not having to catch her up every single time we talk. She knew where we were; that was incredibly helpful.

Center staff agreed that a helpful feature of the coaching was that it offers “tailored navigation through the tools and resources available” that is “customized to whatever state needs dictate.” They noted that a success factor is the coaches’ "interpersonal expertise” and that teams have “a dedicated coach … [resulting in] a rich relationship that supports and connects teams over time to Center resources” and adapt to teams’ evolving needs.

## Critique of the Cohort Program

Many participants commented that the LI was very intensive and taught too many concepts in a short period of time. This led to inadequate time for teams “to process and apply what we have learned” to challenges and “to reflect and let [a concept] sink in at the moment it's taught,” as participants put it. Relatedly, many teams wished for additional time with coaches to help them apply the material to the challenges and plan their approach. Others asked for more time to rest. The staff responded to these suggestions by greatly decreasing the amount of content addressed in later cohorts. Others recommended increasing opportunities to network with other teams and simplifying the Cohort Program’s learning management system, among other technical concerns. A few participants suggested that the staff could reduce the LI’s intensity by providing readings or webinars on key concepts before the LI so that more time at the LI could be given to application. In the focus group, the staff made several suggestions that the program took up, including moving some content to short asynchronous modules for use after the LI by participants and their teams, and structuring shorter synchronous skill-training institutes that participants could use to review the material or promote to their teams.

## Discussion

Participants and staff believe that the Cohort Program effectively merges a practical skill-based curriculum, work-based team learning, and coaching. The LI provides skills and tools with opportunities for practice, strengthens teams’ relationships with their coach, and builds the team. Subsequent work-based learning provides a lengthy “practice space” for teams to think through their challenge and creatively apply skills to address it. Teams value close relationships with their coach, who provides accessible and tailored guidance in navigating team relationships and their challenge.

This article contributes to the literature by describing in detail a program that helps participants learn to use in the workplace skills that are essential for improving PH systems today, including systems thinking and integration, evidence-based decision-making and implementation, change management, adaptive leadership, and improving equity in services and outcomes (Public Health Leadership Forum, 2020; AMCHP, 2016). This study also provides more details than most prior studies (Orton et al., 2006; Umble et al., 2012) about how a program concretely structures evidence-supported methods, such as enrolling teams; teaching interactively (Kane, 2012); offering practical tools to help participants grasp and apply skills in the worksite; integrating a sequence of learning activities rather than relying only on classroom experience; offering opportunities to practice skills in the workplace; providing workplace coaching to help participants experience the skills in practice and overcome barriers; and enrolling teams (Davis et al., 1999; Institute of Medicine, 2010; Moore et al., 2009). The article’s qualitative data explains how learners experience the benefits of such methods, an “inside view” that few prior evaluations have presented.

The Cohort Program also benefited from expert facilitators and coaches with substantial PH experience, allowing teams to choose their challenge and design their approach to addressing it, and extensive improvement-oriented evaluation.

One limitation of this evaluation is that it relies solely on the self-report of participants and staff, without independent verification of the comments. However, this limited process evaluation will be supplemented by evaluations of the program’s impacts on leaders and their initiatives (Coffey et al., in press). Much of this evaluation’s improvement-oriented data was gathered after the Learning Institute; future process evaluations could ask more systematically at the program’s conclusion for participant input about ways to improve it. Additionally, efforts could be made to ask for participant feedback at multiple time points throughout program implementation. Future evaluations should also more formally record staff views on program strengths and areas for improvement, such as the focus group notes that this evaluation used; Cohort Program leaders have quite often, but mostly informally, sought and used staff ideas to improve the program. Staff focus group participants may have hesitated to share areas for program improvement since Center leaders were present, but we think staff felt free to offer corrective feedback because the program culture encouraged transparency and continuous improvement.

Too often, continuing professional development programs still rely on the “update model” described by Nowlen (1988), in which learners individually enroll and receive large amounts of content in a one-day training event with no coaching or follow-up. Future professional development programs for MCH professionals should creatively integrate the practices that this article describes as they recruit and select teams of participants, structure curricula, assemble and integrate learning methods, and design opportunities to apply skills in the workplace. The form of “action learning” described here—including work-based learning in teams, a multi-day Learning Institute and coaching—offers a comprehensive and evidence-based approach to contemporary MCH workforce development.

## Data Availability

Not applicable.

## Footnotes

1 A more extensive version of this article may be found at https://mchwdc.unc.edu/wp-content/uploads/sites/20881/2021/03/Final-White-Paper-Process-Eval-March-2021.pdf.

2 The Maternal and Child Health Bureau, which is part of the federal Health Resources and Services Administration, uses the Title V MCH Block Grant Program to fund MCH programs in states and jurisdictions to improve health outcomes for mothers, infants, and children nationwide. The “Title V workforce” refers to staff members of these funded MCH programs, who mostly work at the state level. For more information, see https://mchb.hrsa.gov/programs-impact/title-v-maternal-child-health-mch-block-grant.
